# Evaluation of Allele-Specific Somatic Changes of Genome-Wide Association Study Susceptibility Alleles in Human Colorectal Cancers

**DOI:** 10.1371/journal.pone.0037672

**Published:** 2012-05-21

**Authors:** Madelyn M. Gerber, Heather Hampel, Nathan P. Schulz, Soledad Fernandez, Lai Wei, Xiao-Ping Zhou, Albert de la Chapelle, Amanda Ewart Toland

**Affiliations:** 1 Integrated Biomedical Sciences Graduate Program, The Ohio State University, Columbus, Ohio, United States of America; 2 The Ohio State University College of Medicine, Columbus, Ohio, United States of America; 3 Division of Human Genetics, Department of Internal Medicine, The Ohio State University, Columbus, Ohio, United States of America; 4 Human Cancer Genetics Program, Comprehensive Cancer Center, The Ohio State University, Columbus, Ohio, United States of America; 5 The Center for Biostatistics, The Ohio State University, Columbus, Ohio, United States of America; 6 Department of Pathology, The Ohio State University, Columbus, Ohio, United States of America; 7 Department of Molecular Virology, Immunology and Medical Genetics, The Ohio State University, Columbus, Ohio, United States of America; National Cancer Institute, National Institutes of Health, United States of America

## Abstract

**Background:**

Tumors frequently exhibit loss of tumor suppressor genes or allelic gains of activated oncogenes. A significant proportion of cancer susceptibility loci in the mouse show somatic losses or gains consistent with the presence of a tumor susceptibility or resistance allele. Thus, allele-specific somatic gains or losses at loci may demarcate the presence of resistance or susceptibility alleles. The goal of this study was to determine if previously mapped susceptibility loci for colorectal cancer show evidence of allele-specific somatic events in colon tumors.

**Methods:**

We performed quantitative genotyping of 16 single nucleotide polymorphisms (SNPs) showing statistically significant association with colorectal cancer in published genome-wide association studies (GWAS). We genotyped 194 paired normal and colorectal tumor DNA samples and 296 paired validation samples to investigate these SNPs for allele-specific somatic gains and losses. We combined analysis of our data with published data for seven of these SNPs.

**Results:**

No statistically significant evidence for allele-specific somatic selection was observed for the tested polymorphisms in the discovery set. The *rs6983267* variant, which has shown preferential loss of the non-risk T allele and relative gain of the risk G allele in previous studies, favored relative gain of the G allele in the combined discovery and validation samples (corrected p-value = 0.03). When we combined our data with published allele-specific imbalance data for this SNP, the G allele of *rs6983267* showed statistically significant evidence of relative retention (p-value = 2.06×10^−4^).

**Conclusions:**

Our results suggest that the majority of variants identified as colon cancer susceptibility alleles through GWAS do not exhibit somatic allele-specific imbalance in colon tumors. Our data confirm previously published results showing allele-specific imbalance for *rs6983267*. These results indicate that allele-specific imbalance of cancer susceptibility alleles may not be a common phenomenon in colon cancer.

## Introduction

Tumor suppressor genes and oncogenes have long been recognized to show copy number losses and gains in tumors, respectively [1], [2]. Classically, the wild-type allele of tumor suppressor genes is lost in tumors whereas the mutated or non-functional allele shows selective retention. Likewise, an activated mutation or activated copy of an oncogene is frequently selected for gain or amplification in tumors. Previous studies using mouse models show evidence that a subset of susceptibility loci for skin and colon cancer demonstrate strain-specific gains or losses consistent with these loci housing tumor promoting alleles or tumor suppressing alleles [3], [4]. For example, *PTPRJ*, a gene originally identified as a candidate tumor suppressor mapping to the mouse *Scc1* locus, was shown to preferentially lose a suspected resistance allele in a subset of heterozygous human colorectal adenocarcinomas showing loss of heterozygosity at *PTPRJ* [3]. Allele-specific gains of a single nucleotide polymorphism (SNP) in *AURKA*, *rs2273535*, have been observed in multiple studies of colorectal tumors [5], [6]. Preferential allelic gains or losses in multiple regions of the genome have been identified in genome-wide screens looking at individuals with multiple independent primary tumors [7] and in genomic studies of glioblastoma samples via the comparison of germline and somatic genotype data [8].

Several genome-wide association studies have revealed alleles associated with colorectal cancer (CRC) risk [9]–[16]. The SNP *rs6983267* on *8q24* has been associated with both colorectal and prostate cancer risk at a genome-wide significance level [9], [17], [18]. Allele-specific copy number analyses showed that the G allele (the putative risk allele) of this variant shows preferential gains in colon tumors and myeloid leukemia [19]–[21]. To our knowledge, no other SNPs from published GWAS literature have definitively and reproducibly shown allele-specific imbalance in colorectal tumors, although individual studies have described allelic imbalance in CRC for other loci [7], [22]. In the present study, we performed quantitative genotyping of 16 statistically significant variants from published GWAS (including *rs6983267*) in paired normal and colorectal tumor DNA. The goal of this study was to investigate these SNPs for somatic gain of the susceptibility allele or loss of the resistance allele using allelic imbalance analyses.

## Methods

### Human Samples

#### Ethics statement

This study was approved by The Ohio State University (OSU) Institutional Review Board. All study participants provided written informed consent for use of their tissues in research.

#### Discovery Set

Paired normal and formalin-fixed paraffin-embedded (FFPE) tumor tissue blocks were obtained through the OSU Human Tissue Research Network and the Midwest Cooperative Human Tissue Network. Tumors that exhibited microsatellite stability and/or stained positively for the Lynch syndrome proteins MSH2, MLH1, PMS2, and MSH6 by immunohistochemistry (IHC) were prioritized for inclusion in the study. When microsatellite or IHC data were unavailable, tumors that showed characteristics suggestive of Lynch syndrome such as right-sided location, poor differentiation, and a high percentage of mucin were excluded [23]. After selection, confirmation of diagnosis and DNA extraction, 194 histologically normal/tumor DNA pairs were available for study.

#### Validation Set

A validation set of 296 paired non-tumor/tumor DNA samples were obtained from two existing study collections. Samples from 196 individuals were acquired from a population-based study cohort of incident colon cancer diagnosed in the metropolitan Columbus area [24], [25]. Blood DNA was available for all cases. An additional 100 fresh frozen paired normal and tumor tissue samples were obtained through the Cooperative Human Tissue Network at The Ohio State University Medical Center. Specimens were snap-frozen shortly after surgery and received anonymously along with a full pathology report. The 296 CRC cases were all classified as likely to be microsatellite stable, the set of 196 samples was stable by microsatellite instability testing, and the 100 fresh frozen tumors all showed intact mismatch repair proteins by immunohistochemistry staining.

### DNA Extraction

#### Test Set

Hematoxylin and eosin stains from normal and tumor FFPE sections were evaluated by a pathologist to confirm diagnosis and to mark tissues for coring. Tissue cores of 1.6 mm diameter were prepared from regions consisting of 70% or more tumor cells for collection of tumor DNA, or from regions with normal histology for isolation of normal (non-tumor) DNA. Genomic DNA was extracted from tissue cores as previously described [26] and quantified with a Nanodrop-1000 spectrophotometer. The majority of DNAs were of good quality as indicated by A260/A280 ratios greater than 1.8.

#### Validation Set

Tumor DNAs from the Columbus-metropolitan area study were isolated as described [26]. Normal DNAs from these individuals were isolated from blood samples in the OSU Human Genetics Sample Bank by standard protocols. DNAs from the 100 paired normal/tumor DNA samples from the Cooperative Human Tissue Network were isolated from the fresh frozen tissue by the same extraction protocol used for the test set samples. Normal DNAs from the three sources (FFPE, blood, and fresh frozen tissue) exhibited similar frequencies of heterozygosity and similar A260/A280 ratios, suggesting comparable DNA quality across sample sources.

### Inclusion of SNPs for Study

To test our hypothesis that CRC susceptibility loci would show allele-specific somatic events in tumors, we searched the recent literature to identify variants showing evidence of CRC risk from GWA studies [9], [10], [13]–[15], [27]–[32]. Seventeen SNPs (*rs10411210*, *rs10936599*, *rs11169552*, *rs16892766*, *rs3802842*, *rs4444235*, *rs4779584*, *rs4925386*, *rs4939827*, *rs6687758*, *rs6691170*, *rs6983267*, *rs7014346*, *rs7136702*, *rs719725*, *rs961253*, *rs9929218*) meeting or approaching genome-wide significance (p-value<10^−7^) for CRC risk in published GWA studies were chosen for analysis of allele-specific imbalance in the initial discovery set of tumor/normal DNA pairs (Table 1). Other inclusion criteria for study included identification in Caucasian populations and a sufficiently high documented minor allele frequency (MAF>20%) for identification of enough heterozygotes for statistical power. The SNP *rs16892766* was the only exception to this criterion, as it has a documented MAF of 7%. *rs4925386* was eliminated post-genotyping of the original sample set due to a failure rate greater than 15%.

**Table 1:** CRC risk-associated GWAS SNPs assessed for allele-specific imbalance in the present study.

SNP	Position*	Gene(s)/Locus	Genotype	dbSNP MAF†	GWAS Reference(s)	OR (95% CI)§	P-value‡
*rs10411210*	chr19:33,532,300	*RHPN2*	CT	T = 0.21	[10]	0.87 (0.83–0.91)	4.6×10^−9^
*rs10936599*	chr3:169,492,101	*MYNN* synonymous coding	CT	T = 0.30	[15]	0.93 (0.91–0.96)	3.39×10^−8^
*rs11169552*	chr12:51,155,663∞	*ATF1*, *DIP2B*	CT	T = 0.24	[15]	0.92 (0.90–0.95)	1.89×10^−10^
*rs16892766*	chr8:117,630,683	*EIF3H*	AC	C = 0.07	[14], [32]	1.43 (1.13–1.82)	3.3×10^−18^
*rs3802842*	chr11:111,171,709	*C11orf93*	AC	C = 0.31	[13], [32]	1.11 (1.08–1.15)	5.8×10^−10^
*rs4444235*	chr14:54,410,919	*BMP4*	TC	C = 0.44	[10], [32]	1.11 (1.08–1.15)	8.1×10^−10^
*rs4779584*	chr15:32,994,756	*SCG5*, *GREM1*	CT	T = 0.46	[14], [32]	1.70 (1.35–2.14)	4.7×10^−7^
*rs4925386*	chr20:60,921,044	*LAMA5*	CT	T = 0.41	[15]	0.93 (0.91–0.95)	1.89×10^−10^
*rs4939827*	chr18:46,453,463	*SMAD7*	CT	T = 0.38	[27], [14], [13], [32]	0.85 (0.81–0.89)	1.0×10^−12^
*rs6687758*	chr1:222,164,948#	Intergenic	AG	G = 0.22	[15]	1.09 (1.06–1.12)	2.27×10^−9^
*rs6691170*	chr1:222,045,446#	Intergenic	GT	T = 0.26	[15]	1.06 (1.03–1.09)	9.55×10^−10^
*rs6983267*	chr8:128,413,305▵	Intergenic	GT	T = 0.44	[9], [28], [14], [31], [32]	1.21 (1.15–1.27)	1.27×10^−14^
*rs7014346*	chr8:128,424,792▵	Intergenic	GA	A = 0.33	[13]	1.19 (1.15–1.23)	8.6×10^−26^
*rs7136702*	chr12:50,880,216∞	*LARP4*, *DIP2B*	CT	T = 0.46	[15]	1.06 (1.04–1.08)	4.02×10^−8^
*rs719725*	chr9:6,365,683	Intergenic	AC	C = 0.33	[29], [28], [30]	1.13 (NA)	4.98×10^−5^
*rs961253*	chr20:6,404,281	*BMP2*	CA	A = 0.29	[10], [32]	1.12 (1.08–1.16)	2.0×10^−10^
*rs9929218*	chr16:68,820,946	*CDH1*	GA	A = 0.25	[10], [32]	0.91 (0.89–0.94)	1.2×10^−8^

* Position by UCSC Genome Browser (Human Feb. 2009 GRCh37/hg19 Assembly).

† MAF = Minor Allele Frequency, as listed on NCBI dbSNP.

§ Odds Ratio from first listed GWAS reference (95% confidence interval). NA = not available.

‡ P-value from first listed GWAS reference.

∞ SNP positions in modest linkage disequilibrium (D′ = 0.76, ref. 15).

# SNP positions in modest linkage disequilibrium (D′ = 0.71, ref. 15).

▵ SNP positions in high linkage disequilibrium (D′ = 0.99, ref. 9).

### Quantitative Genotyping

Multiplexed primers for PCR amplification and allele-specific single base extension reactions were designed using the Sequenom® MassARRAY Assay Design 3.1 software and are available upon request. Mass spectrometry-based genotyping of 20 ng paired tumor and normal DNA was performed using Sequenom® MassARRAY iPlex Gold (Sequenom Inc., San Diego, CA, USA) according to the manufacturer's protocol. Each 384-well Sequenom® plate included four negative template controls (dH_2_O), two samples tested in duplicate, and four positive control DNAs.

### Verification of Genotyping Technique

To validate the use of Sequenom® quantitative genotyping for its sensitivity for identification of allelic imbalance, we generated natural log-transformed N-ratios (N-ratio = normal allele 1 peak area/normal allele 2 peak area) for DNA mixtures of known homozygote DNA samples representing 0, 20, 40, 50, 60, 80, and 100% allelic contributions. We did not have appropriate homozygous DNAs for three of the SNPs so these were not evaluated. The majority of slopes and R-values for these were very close to standard curves for “perfect data” suggesting a high degree of sensitivity for our method of detecting allelic deviations from 50% (Figure S1).

### Analysis of Imbalance

The Sequenom® MassARRAY iPLEX software quantifies the area under each of the allele peaks and assigns either a heterozygous or homozygous call to the SNP by calculating the ratio of the peak areas for the two alleles. As described previously [7], for all SNPs tested we scored preferential allelic imbalance by calculating the R-ratio for each DNA pair. We defined the R-ratio as the ratio of the two allele peak areas in the normal DNA divided by the ratio of the two allele peak areas in the paired tumor DNA (R-ratio = Normal^(allele 1/allele 2)^/Tumor^(allele 1/allele 2)^). Samples were scored as having imbalance, defined as the loss of either the first or second allele in the tumor sample, if the R-ratio was greater than 1.5 or less than 0.67, respectively. The R-ratio thresholds used to determine imbalance have been described previously [33], [34]. A chi-squared test (df = 1) was used to assess the observed imbalances for statistically significant deviation from the expected 50∶50 distribution of allele imbalances. In cases in which a tumor was heterozygous for a SNP by genotyping but the paired normal sample failed to genotype, an average of the two normal alleles for heterozygous normal samples at that SNP was used in place of the failed normal sample to calculate an R-ratio. SNPs with p-value<0.10 were considered suggestive of preferential allelic imbalance and were therefore subjected to testing in the validation sample set to rule out false positives. Bonferroni correction was used to adjust for the number of statistical tests. In addition to qualitative determination of imbalance, we generated box plots of the distribution of R-ratios for each SNP for samples showing relative loss of allele 1, relative loss of allele 2, and no imbalance (Figure S2). Samples were excluded from the plots if they had an R-ratio of greater than 10 or if an R-ratio could not be calculated because one of the two alleles in the tumor sample had an allele peak area value of 0.

### Validation Studies

Following statistical analysis of allele-specific imbalance in the discovery sample set, three variants with p-values<0.1 (*rs16892766*, *rs6983267* and *rs7136702*) were genotyped by Sequenom® MassARRAY iPlex Gold in a replication sample set of 296 paired normal/tumor DNAs. The same quantitative genotyping protocol and statistical analyses used for the discovery sample set were employed with the validation sample set. Bonferroni correction was used to adjust for the number of statistical tests (n = 3).

### Compilation of Allele-Specific Imbalance Data from Multiple Studies

Allele-specific imbalance analyses have previously been performed on seven of the GWAS SNPs tested in the present study [19], [35]. These studies employed manual measurement of sequencing chromatogram peaks for tumor and normal DNAs to calculate R-ratios. Both published studies utilized R-ratio cutoff values of <0.60 and >1.67 for allele-specific imbalance analysis. For both previously published studies, tumor DNA was isolated from fresh frozen colon tumors, and blood was used as the source of normal DNA [19], [35]. In order to test the seven variants that overlapped with our study, we combined the data from the published studies with our allele-specific imbalance results for *rs6983267*, *rs961253*, *rs3802842*, *rs10411210*, *rs4444235*, *rs4779584*, and *rs9929218*. We combined our numbers of relative allelic losses with the numbers from the published studies and performed a chi-squared test with Bonferroni correction (n = 7) to determine the statistical significance of the combined imbalances.

### Correlation Analysis of Allelic Imbalances and Age, Sex, and Tumor Stage

For each SNP successfully assessed for allelic imbalance, we investigated the association between the presence of allelic imbalance and age of diagnosis, sex, and tumor stage of the patient. Chi-squared statistical test was used to detect association between allelic imbalance and sex. Fisher exact statistical test was used to detect association between allelic imbalance and tumor stage. For tumor stage, we classified tumors as TNM stage I–IV according to available tumor size, nodal spread, and metastasis information. The sample t-test was used to compare the average age of patients whose tumors showed allelic imbalance to that of patients whose tumors maintained heterozygosity. Correlations with corrected p-values<0.05 were deemed statistically significant.

## Results

### Discovery Set Genotyping

To determine if any of the 17 CRC-associated SNPs show evidence of allele-specific imbalance, we genotyped them in 194 normal/tumor DNA pairs. All but one SNP, *rs4925386*, were successfully genotyped in greater than 85% of samples in the discovery set. Due to a high rate of genotyping failures (24%), *rs4925386* was excluded from further analysis. The number of heterozygous normal DNAs identified for each SNP (for which the paired tumor DNA was also successfully genotyped) ranged from 27 to 84 of the 194 samples (14–43%; Table 2). The frequency of overall relative allele loss (for both risk and non-risk alleles combined) ranged from 2% to 44%. While none of the SNPs reached statistical significance for allele-specific imbalance at α = 0.05, three SNPs (*rs16892766, rs6983267, rs7136702*) showed a trend for allele-specific imbalance (p-values<0.10) prior to Bonferroni correction for multiple comparison (n = 16). The SNP *rs6983267* showed higher frequencies of relative loss of the non-risk T allele compared to the risk G allele. Interestingly, *rs16892766* and *rs7136702* both demonstrated higher frequencies of relative loss of the risk allele compared to the non-risk allele in the discovery set tumors. The variants *rs16892766*, *rs6983267* and *rs7136702* were prioritized for validation in a second set of samples. In addition to qualitatively scoring the SNPs as showing imbalance or no imbalance, the distribution of R-ratios for relative loss of the risk allele, relative loss of the non-risk allele and no imbalance were graphed as boxplots for each SNP (Figure S2). Samples for which the R-ratio was greater than 10 or for which the R-ratio could not be calculated were excluded from the plots.

**Table 2:** Analysis of allele-specific imbalance in discovery sample set.

SNP	Risk Allele	Non-risk Allele	Risk Allele Lost*	Non-risk Allele Lost†	Total Imbalance§	Unadjusted P-value‡
*rs16892766*	C	A	6 (22%)	1 (4%)	7/27 (26%)	0.06
*rs6983267*	G	T	6 (9%)	14 (21%)	20/67 (30%)	0.07
*rs7136702*	T	C	12 (16%)	5 (7%)	17/75 (23%)	0.09
*rs10936599*	C	T	6 (12%)	2 (4%)	8/49 (16%)	0.16
*rs3802842*	C	A	9 (12%)	4 (5%)	13/75 (17%)	0.17
*rs961253*	A	C	11 (15%)	6 (8%)	17/71 (24%)	0.23
*rs6687758*	G	A	8 (15%)	4 (7%)	12/55 (22%)	0.25
*rs4779584*	T	C	9 (17%)	14 (26%)	23/53 (43%)	0.30
*rs4939827*	T	C	19 (24%)	15 (19%)	34/78 (44%)	0.49
*rs7014346*	A	G	6 (7%)	4 (5%)	10/82 (12%)	0.53
*rs9929218*	G	A	2 (3%)	1 (2%)	3/62 (5%)	0.56
*rs10411210*	C	T	4 (11%)	3 (9%)	7/35 (20%)	0.71
*rs4444235*	C	T	8 (10%)	7 (9%)	15/81 (19%)	0.80
*rs719725*	A	C	8 (12%)	9 (14%)	17/65 (26%)	0.81
*rs6691170*	T	G	5 (6%)	5 (6%)	10/79 (13%)	1.00
*rs11169552*	C	T	1 (1%)	1 (1%)	2/84 (2%)	1.00

* *Risk Allele Lost* refers to relative loss of the risk allele compared to the non-risk allele. Number in parentheses indicates percentage of total heterozygous samples showing relative loss of risk allele.

† *Non-risk Allele Lost* refers to relative loss of the non-risk allele compared to the risk allele. Number in parentheses indicates percentage of total heterozygous samples showing relative loss of non-risk allele.

§ Total number of tumors with imbalance/total heterozygous samples (% of heterozygotes showing imbalance).

‡ Chi-squared statistical test, df = 1. Unadjusted for multiple comparisons.

### Validation Set Genotyping

The SNPs *rs16892766*, *rs6983267* and *rs7136702*, which all showed evidence of allele-specific imbalance in the original discovery set, were further tested in the validation sample set of 296 normal/tumor DNA pairs. As with the test set, these three SNPs successfully genotyped in greater than 85% of the validation samples. With 22% of the validation set heterozygotes showing relative loss of an allele, *rs6983267* showed a frequency of overall relative allele loss lower than that observed in the original test set (30%; Table 3). A lower frequency of heterozygous samples in the validation set showed relative loss of an allele of *rs7136702* (11%) compared to the test set (23%; Table 3). Similarly, a lower frequency of allelic loss of *rs16892766* was observed in the validation sample set (16%) compared to the original test set (26%; Table 3). *rs6983267* again showed a tendency towards statistically significant preferential allelic imbalance (p-value = 0.06), favoring relative loss of the non-risk T allele and relative retention of the risk G allele in the validation sample set. However, neither *rs7136702* nor *rs16892766* showed a statistically significant tendency towards preferential allelic imbalance in the validation sample set (p-values = 0.59 and 1.00, respectively).

**Table 3:** Analysis of allele-specific imbalance in discovery, validation, and combined sample sets.

SNP	Sample Set	Risk Allele	Non-risk Allele	Risk Allele Lost*	Non-risk Allele Lost†	Total Imbalance§	P-value‡	Adjusted P-value∞
*rs7136702*								
	Discovery	T	C	12 (16%)	5 (7%)	17/75 (23%)	0.09	1.00
	Validation	T	C	6 (5%)	8 (6%)	14/133 (11%)	0.59	1.00
	Combined	T	C	18 (9%)	13 (6%)	31/208 (15%)	0.37	1.00
*rs16892766*								
	Discovery	C	A	6 (22%)	1 (4%)	7/27 (26%)	0.06	0.96
	Validation	C	A	3 (8%)	3 (8%)	6/38 (16%)	1.00	1.00
	Combined	C	A	9 (14%)	4 (6%)	13/65 (20%)	0.17	0.51
*rs6983267*								
	Discovery	G	T	6 (9%)	14 (21%)	20/67 (30%)	0.07	1.00
	Validation	G	T	9 (7%)	19 (15%)	28/125 (22%)	0.06	0.18
	Combined	G	T	15 (8%)	33 (17%)	48/192 (25%)	0.01	0.03

* *Risk Allele Lost* refers to relative loss of risk allele compared to non-risk allele. Number in parentheses indicates percentage of total heterozygous samples showing relative loss of risk allele.

† *Non-risk Allele Lost* refers to relative loss of non-risk allele compared to risk allele. Number in parentheses indicates percentage of total heterozygous samples showing relative loss of non-risk allele.

§ Total number of tumors with imbalance/total heterozygous samples (% of heterozygotes showing imbalance).

‡ Chi-squared statistical test, df = 1.

∞ Bonferroni correction for 16 multiple comparisons (original) or 3 multiple comparisons (validation, combined).

### Combined Genotyping Results from Discovery and Validation Sample Sets

When the test set and validation set genotypes were combined, 48 of 192 heterozygous samples (25%) showed relative loss of an allele of *rs6983267* (Table 3). For the SNP *rs7136702*, 31 of 208 combined heterozygotes showed relative loss of either allele (15%). When genotypes from the test set and validation set were combined for *rs16892766*, 13 of 65 heterozygotes (20%) showed allelic loss. By pooled analysis *rs6983267* showed strong statistical evidence of preferential allelic imbalance (p-value = 0.01). After Bonferroni correction for multiple comparisons testing (n = 3), *rs6983267* maintained a statistically significant adjusted p-value of 0.03. In contrast, both *rs16892766* and *rs7136702* failed to show any tendency towards significant allele-specific imbalance by combined analysis (unadjusted p-values = 0.17 and 0.37, respectively).

### Compilation of Allelic Imbalance Data from Multiple Studies

Because others have published allele-specific imbalance data on seven variants from our study [19], [35], we decided to perform combined analysis of the present study and the previously published studies to increase the power of identifying SNPs demonstrating allele-specific imbalance. When the imbalances observed in our samples at the SNPs *rs6983267*, *rs961253*, *rs3802842*, *rs10411210*, *rs4444235*, *rs4779584*, and *rs9929218* were combined with those published previously [19], [35], we observed a highly significant relative loss of the non-risk T allele of *rs6983267* (p-value = 2.94×10^−5^). After Bonferroni correction (n = 7), the preferential relative loss of the T allele of *rs6983267* maintained a highly significant p-value of 2.06×10^−4^. None of the other variants showed statistically significant evidence of preferential allelic imbalance (Table 4).

**Table 4:** Combined analysis with published allele-specific imbalance studies.

SNP	Risk Allele	Non-risk Allele	Risk Allele Lost/Total Hets* † §	Risk Allele Lost/Total Hets (OSU)§	Total Risk Allele Lost	Non-risk Allele Lost/Total Hets* † §	Non-risk Allele Lost/Total Hets (OSU)§	Total Non-risk Allele Lost	P-value‡	Adjusted P-value∞
*rs6983267*	G	T	34/466 (7%)*	15/192 (8%)	49	67/466 (14%)*	33/192 (17%)	100	2.94×10^−5^	2.06×10^−4^
*rs961253*	A	C	16/88 (18%)†	11/71 (15%)	27	11/88 (13%)†	6/71 (8%)	17	0.13	0.92
*rs3802842*	C	A	5/89 (6%)†	9/75 (12%)	14	4/89 (4%)†	4/75 (5%)	8	0.20	1.00
*rs10411210*	C	T	5/174 (3%)†	4/35 (11%)	9	10/174 (6%)†	3/35 (9%)	13	0.39	1.00
*rs4444235*	C	T	10/90 (11%)†	8/81 (10%)	18	7/90 (8%)†	7/81 (9%)	14	0.48	1.00
*rs4779584*	T	C	9/87 (10%)†	9/53 (17%)	18	8/87 (9%)†	14/53 (26%)	22	0.53	1.00
*rs9929218*	G	A	4/90 (4%)†	2/62 (3%)	6	6/90 (7%)†	1/62 (2%)	7	0.78	1.00

* Allelic imbalance data from [19].

† Allelic imbalance data from [35].

§ Percentage indicates proportion of heterozygotes with allelic imbalance.

‡ Chi-squared statistical test, df = 1. Unadjusted for multiple comparisons.

∞ Bonferroni correction for 7 multiple comparisons.

### Correlation Analysis of Allelic Imbalances and Age, Sex, and Tumor Stage

To test whether samples showing allelic imbalance for the GWAS SNPs had different clinical characteristics compared to samples not showing imbalance, we performed a correlation analysis of imbalance with age, sex and tumor stage using data from our discovery sample set. The presence of allelic imbalance was significantly associated with tumor stage for *rs719725* (unadjusted p-value = 0.0098), and significantly associated with younger age for *rs7014346* (unadjusted p-value = 0.033). However, after adjusting for multiple comparisons (n = 16), there was no significant association between the presence of allelic imbalance and age, sex, and tumor stage (adjusted p-values>0.05) for any of the tested SNPs.

## Discussion

In this study, we investigated 16 SNPs previously associated with CRC risk for allele-specific imbalance using the Sequenom® MassARRAY iPLEX Gold genotyping platform. While 15 of the 16 tested SNPs did not show statistically significant evidence (p-value<0.05) of preferential allelic imbalance in our discovery sample set, the SNP *rs6983267* demonstrated a tendency towards statistically significant somatic loss of the non-risk T allele and retention of the risk G allele in both the original discovery set and the validation sample set (p-values = 0.07 and 0.06, respectively; Tables 2 and 3). This is consistent with previously published reports [19], [20]. Interestingly, despite being in high linkage disequilibrium with *rs6983267* at 8q24 (D′ = 0.99) [9], [13], *rs7014346* did not show evidence of preferential allelic imbalance (p-value = 0.53) in the discovery sample set. In the largest previous study to assess allelic imbalance for *rs6983267*, 466 heterozygous tumors from Finnish CRC patients were successfully evaluated and 101 of these heterozygous samples (22%) showed allelic imbalance [19]. Among these 101 samples, there were significantly (p-value = 0.0007) more tumors showing relative loss of the T allele (66% of tumors) versus relative loss of the G allele (34% of tumors). From our discovery and validation sets combined, we evaluated tumors from individuals heterozygous for the *rs6983267* variant, and 48 (25%) of these heterozygotes showed allelic imbalance. We observed a nearly identical percentage of tumors showing relative loss of the T allele (33 of 48; 69%) versus the G allele (15 of 48; 31%). This was significant even after adjusting for multiple comparisons testing (p-value = 0.03; Table 3). Thus, our data support the observation of preferential allelic imbalance for *rs6983267* and validate our experimental method. Furthermore, when we combined our data with that of Tuupanen et al. [19], we observed a highly significant relative loss of the T allele and relative gain of the G allele that withstood multiple comparisons testing (p-value = 2.06×10^−4^; Table 4). Importantly, the finding that the risk G allele may be selectively retained or gained in colorectal tumors is consistent with a study showing that the G allele of *rs6983267* demonstrates enhanced binding of the Wnt-regulated transcription factor TCF4, perhaps leading to increased responsiveness to Wnt signaling in individuals carrying the G risk allele [20]. Additionally, these data confirm that allele-specific imbalance does occur for CRC susceptibility loci, albeit at a low frequency.

In another recent study, somatic allelic imbalance was investigated at seven low-penetrance CRC susceptibility loci [35]. The loci-tagging SNPs *rs4779584*, *rs3802842*, *rs4444235*, *rs9929218*, *rs10411210*, and *rs961253* that were genotyped in our study were among the seven variants tested for allele specific imbalance in the study by Niittymäki et al. [35]. While none of these SNPs showed evidence of preferential allelic imbalance in the combined analysis with our data, one of these SNPs (*rs961253*) demonstrated similar allelic imbalance trends as those observed in our discovery sample set, with *rs961253* showing more frequent relative loss of the A allele in both studies (Table 4). Rates of heterozygosity and imbalance were very similar between the two studies with the exception of our study showing a higher degree of allelic imbalance for *rs4779584*.A combined analysis of our data and the data from Niittymäki et al. [35] for the six variants in common did not reveal any SNPs with evidence of allele-specific imbalance. A caveat to combining data from the present study with that from published data sets is that the percentage of tumor cells in the samples as well as genotyping methods and R-value cutoffs for determining allelic imbalance differ across studies. Nonetheless, our study reproduces the finding that these six loci-tagging SNPs show no evidence for preferential allelic imbalance in predominantly Caucasian study populations.

Although only one of the SNPs tested in the present study showed strong evidence of preferential allelic imbalance, the other SNPs may play a role in germline predisposition for CRC independent of somatic events in the tumor. It has been proposed that these SNPs influence the development of neoplasms but do not affect subsequent somatic neoplastic progression [35]. The functional SNPs at the GWAS-identified loci may influence neoplastic development by modifying gene expression, methylation, or splicing patterns in such a way that selection at the DNA level is not required during tumorigenesis. These SNPs could also impact non-tumor cells, such as stromal or immune cells to modify cancer risk, but be independent of the cancer cells themselves. Once the mechanism by which these variants act to confer risk is better understood, we may be able to deduce which variants are more likely to show selection in tumors.

Inherent limitations in our study design could further mask existing preferential allelic selection. First, it is possible that normal cells were isolated with tumor cells in the tumor tissue cores from which DNA was extracted for analysis. Despite initial selection of regions of the tumor containing 70% or greater tumor cells, some normal DNA contamination of the tumor DNA sample could bias the sample towards showing no imbalance. However, our histological examination of the tissue samples should minimize the possibility of normal DNA contamination. Similarly, our histologically normal samples from FFPE colon tissue may not be normal and may contain similar somatic mutations as the tumor, which could result in a general “undercalling” of tumors with imbalance. Whenever possible the normal colon tissue was collected from sites distant from the tumor. Second, we employed conservative data inclusion practices by discounting aggressive genotype calls made by the Sequenom® MassARRAY iPLEX software and by instilling R-ratio cutoffs of >1.5 and <0.67 for determination of allelic imbalance. Our rigorous requirements for inclusion of data may limit detection of borderline significant allelic imbalance, particularly in tumor samples containing non-tumor cells. Furthermore, if tumors are heterogeneous for allelic loss we may not detect imbalances in that sample. Third, our discovery sample set was limited to 194 normal/tumor DNA pairs and may have lacked statistical power for detection of preferential allelic selection in loci showing lower levels of heterozygosity or less frequent genomic aberration. Based on mouse data showing that about 40% of susceptibility loci demonstrate preferential allelic imbalance [4], we did not expect all SNPs identified through GWA studies to show preferential allelic selection in tumors. However, our results are surprising in that only one SNP, *rs6983267*, showed a trend towards somatic selection in the colon tumors. These results may indicate differences between species, differences between colon and skin tumors, or may be the result of the discussed study limitations.

In conclusion, our results suggest that the majority of variants identified as colon cancer susceptibility alleles through GWAS do not exhibit somatic allele-specific imbalance in colon tumors. However, our data confirm previously published results showing allele-specific imbalance for *rs6983267*. These results indicate that somatic allele-specific imbalance of cancer susceptibility alleles may not be a common phenomenon in colon cancer, but that for a small percentage of loci (1 of 16, or 6%, observed in the present study), somatic selection of specific alleles may be driving tumorigenesis.

## Supporting Information

Figure S1 **Standard Curves for SNPs.** Standard curves for 13 of the tested GWAS SNPs were generated by mixing control DNAs known to be homozygous for either allele in different proportions so as to generate mixtures of 0, 20, 40, 50, 60, 80 and 100% allele 1. DNA mixtures were quantitatively genotyped using Sequenom® MassARRAY iPLEX Gold, and the percentage of allele 1 was plotted against natural log-transformed N-ratio. The line of best fit, linear equation in the form ln(N-ratio) = m(% allele 1)+b, and correlation coefficient R^2^ are shown for each GWAS SNP for which the appropriate control homozygote DNA was available. The expected equation for the ideal standard curve is: ln(N-ratio) = −0.0456(% allele 1)+2.2822.
(DOC)

Figure S2 **Box plots of R-ratios.** Box plots for each call relative loss of allele 1, relative loss of allele 2 and no imbalance are plotted for each of the 16 SNPs genotyped in the discovery sample set. Average R-ratio is indicated by a white line and the standard deviation within each group is denoted. Outlier samples are indicated by a dot except for samples with R-ratios greater than 10 which were removed from the figure.
(DOCX)

## References

[1] Knudson AG (1971). Mutation and cancer: statistical study of retinoblastoma.. Proc Natl Acad Sci USA.

[2] Varmus HE (1984). The molecular genetics of cellular oncogenes.. Ann Rev Genet.

[3] Ruivenkamp CA, van Wezel T, Zanon C, Stassen AP, Vlcek C (2002). Ptprj is a candidate for the mouse colon-cancer susceptibility locus Scc1 and is frequently deleted in human cancers.. Nat Genet.

[4] Nagase H, Mao JH, Balmain A (2003). Allele-specific Hras mutations and genetic alterations at tumor susceptibility loci in skin carcinomas from interspecific mice.. Cancer Res.

[5] Ewart-Toland A, Briassouli P, de Koning JP, Mao JH, Yuan J (2003). Identification of Stk6/STK15 as a candidate low-penetrance tumor-susceptibility gene in mouse and human.. Nat Genet.

[6] Hienonen T, Salovaara R, Mecklin JP, Järvinen H, Karhu A (2006). Preferential amplification of AURKA 91A (Ile31) in familial colorectal cancers.. Int J Cancer.

[7] Dworkin AM, Ridd K, Bautista D, Allain DC, Iwenofu OH (2010). Germline variation controls the architecture of somatic alterations in tumors.. PLoS Genet.

[8] LaFramboise T, Dewal N, Wilkins K, Pe'er I, Freedman ML (2010). Allelic selection of amplicons in glioblastoma revealed by combining somatic and germline analysis.. PLoS Genet.

[9] Tomlinson I, Webb E, Carvajal-Carmona L, Broderick P, Kemp Z (2007). A genome-wide association scan of tag SNPs identifies a susceptibility variant for colorectal cancer at 8q24.21.. Nat Genet.

[10] Houlston RS, Webb E, Broderick P, Pittman AM, Di Bernardo MC (2008). Meta-analysis of genome-wide association data identifies four new susceptibility loci for colorectal cancer.. Nat Genet.

[11] Jaeger E, Webb E, Howarth K, Carvajal-Carmona L, Rowan A (2008). Common genetic variants at the CRAC1 (HMPS) locus on chromosome 15q13.3 influence colorectal cancer risk.. Nat Genet.

[12] Pittman AM, Webb E, Carvajal-Carmona L, Howarth K, Di Bernardo MC (2008). Refinement of the basis and impact of common 11q23.1 variation to the risk of developing colorectal cancer.. Hum Mol Genet.

[13] Tenesa A, Farrington SM, Prendergast JG, Porteous ME, Walker M (2008). Genome-wide association scan identifies a colorectal cancer susceptibility locus on 11q23 and replicates risk loci at 8q24 and 18q21.. Nat Genet.

[14] Tomlinson IP, Webb E, Carvajal-Carmona L, Broderick P, Howarth K (2008). A genome-wide association study identifies colorectal cancer susceptibility loci on chromosomes 10p14 and 8q23.3.. Nat Genet.

[15] Houlston RS, Cheadle J, Dobbins SE, Tenesa A, Jones AM (2010). Meta-analysis of three genome-wide association studies identifies susceptibility loci for colorectal cancer at 1q41, 3q26.2, 12q13.13 and 20q13.13.. Nat Genet.

[16] Xiong F, Wu C, Bi X, Yu D, Huang L (2010). Risk of genome-wide association study-identified genetic variants for colorectal cancer in a Chinese population.. Cancer Epidemiol Biomarkers Prev.

[17] Haiman CA, Le Marchand L, Yamamato J, Stram DO, Sheng X (2007). A common genetic risk factor for colorectal and prostate cancer.. Nat Genet.

[18] Yeager M, Orr N, Hayes RB, Jacobs KB, Kraft P (2007). Genome-wide association study of prostate cancer identifies a second risk locus at 8q24.. Nat Genet.

[19] Tuupanen S, Niittymäki I, Nousiainen K, Vanharanta S, Mecklin JP (2008). Allelic imbalance at rs6983267 suggests selection of the risk allele in somatic colorectal tumor evolution.. Cancer Res.

[20] Tuupanen S, Turunen M, Lehtonen R, Hallikas O, Vanharanta S (2009). The common colorectal cancer predisposition SNP rs6983267 at chromosome 8q24 confers potential to enhanced Wnt signaling.. Nat Genet.

[21] Micale L, Augello B, Daniele G, Macchia G, L'abbate A (2011). Amplification of the G allele at SNP rs6983267 in 8q24 amplicons in myeloid malignancies as cause of the lack of MYC overexpression?. Blood Cells Mol Dis.

[22] Umetani N, Fujimoto A, Takeuchi H, Shinozaki M, Bilchik AJ (2004). Allelic imbalance of *APAF-1* locus at *12q23* is related to progression of colorectal carcinoma.. Oncogene.

[23] Yearsley M, Hampel H, Lehman A, Nakagawa H, de la Chapelle A (2006). Histologic features distinguish microsatellite-high from microsatellite-low and microsatellite-stable colorectal carcinomas, but do not differentiate germline mutations from methylation of the MLH1 promoter.. Hum Pathol.

[24] Hampel H, Frankel WL, Martin E, Arnold M, Khanduja K (2005). Screening for the Lynch Syndrome (hereditary nonpolyposis colorectal cancer).. N Engl J Med.

[25] Hampel H, Frankel WL, Martin E, Arnold M, Khanduja K (2008). Feasibility of screening for Lynch syndrome among patients with colorectal cancer.. J Clin Oncol.

[26] Dworkin AM, Tseng SY, Allain DC, Iwenofu OH, Peters SB (2009). Merkel cell polyomavirus in cutaneous squamous cell carcinoma of immunocompetent individuals.. J Invest Dermatol.

[27] Broderick P, Carvajal-Carmona L, Pittman AM, Webb E, Howarth K (2007). A genome-wide association study shows that common alleles of *SMAD7* influence colorectal cancer risk.. Nat Genet.

[28] Poynter JN, Figueiredo JC, Conti DV, Kennedy K, Gallinger S (2007). Variants on 9p24 and 8q24 are associated with risk of colorectal cancer: results from the colon cancer family registry.. Cancer Res.

[29] Zanke BW, Greenwood CMT, Rangrej J, Kustra R, Tenesa A (2007). Genome-wide association scan identifies a colorectal cancer susceptibility locus on chromosome 8q24.. Nat Genet.

[30] Kocarnik JD, Hutter CM, Slattery ML, Berndt SI, Hsu L (2010). Characterization of 9p24 risk locus and colorectal adenoma and cancer: gene-environment interaction and meta-analysis.. Cancer Epidemiol Biomarkers Prev.

[31] Cui R, Okada Y, Jang SG, Ku JL, Park JG (2011). Common variant in 6q26–q27 is associated with distal colon cancer in an Asian population.. Gut.

[32] Peters U, Hutter CM, Hsu L, Schumacher FR, Conti DV (2011). Meta-analysis of new genome-wide association studies of colorectal cancer risk.. Hum Genet.

[33] Wang C, Horiuchi A, Imai T, Ohira S, Itoh K (2004). Expression of BRCA1 protein in benign, borderline and malignant epithelial ovarian neoplasms and its relationship to methylation and allelic loss of the BRCA1 gene.. J Pathol.

[34] Weber F, Shen L, Fukino K, Patocs A, Mutter GL (2006). Total-genome analysis of BRCA1/2-related invasive carcinomas of the breast identifies tumor stroma as potential landscaper for neoplastic initiation.. Am J Hum Genet.

[35] Niittymäki I, Tuupanen S, Li Y, Järvinen H, Mecklin J-P (2011). Systematic search for enhancer elements and somatic allelic imbalance at seven low-penetrance colorectal cancer predisposition loci.. BMC Medical Genetics.

